# The Retrograde Policy of the Bath Guardians

**Published:** 1914-06-13

**Authors:** Saml. Craddock

**Affiliations:** Summerdale, Bath


*To the Editor of* The Hospital.

### The Retrograde Policy of the Bath Guardians.

*To the Editor of* The Hospital.

Sir,—In your criticism of the Rev. H. Hirst's letter and my own, you state that neither of us challenges a single statement that you have put forward. I took exception to the statement, "That the workhouse infirmary was in such bad repute among the sick poor that they refused to enter it, saying that they would rather die in their own homes." These words are plain English, and pregnant with a denunciation of the infirmary as bad as the imagination can picture, and if true would be a disgrace to everyone connected with its administration and a terrible disgrace to the city of Bath. I reiterate that it is not true, and that there is no hospital or infirmary where the patients are more carefully nursed, better fed, and their general well-being more cared for than at the Bath Infirmary.

During the last winter the fact that the infirmary was nearly always full negatives the reckless assertion of your correspondent. Have Dr. Fuller, Mr. Court, Miss Tod, or Miss Jones reported such a condition, as they would in duty be bound to? If they have, such a report has never been brought to my notice.

I am well aware of the shortcomings of the building itself in regard to the heating, lighting, want of lifts, etc., but these defects tell upon the nursing staff and are not reflected on the patients. I have no wish to discuss at present the bad existing constructive arrangements, as I have no *locus standi* beyond that of being a ratepayer; all I go for in your caustic allegation is the quotation, "*Magna est veritas, et prævalebit.*"—Yours faithfully, Saml. Craddock.

Summerdale, Bath, June 9, 1914.

